# Recent Advance in Regulatory Effect of GRP120 on Bone Metabolism

**DOI:** 10.14336/AD.2023.0216

**Published:** 2023-10-01

**Authors:** Yuhan Wang, Haixia Liu, Zhiguo Zhang

**Affiliations:** Institute of Basic Theory for Chinese Medicine, Chinese Academy of Chinese Medical Sciences, Beijing, China.

**Keywords:** bonem metabolism, GPR120, fatty acids

## Abstract

The link between fatty acids and bone metabolism is complex and can be direct and indirect. This link has been reported in different types of bone cells and various stages of bone metabolism. G-protein coupled receptor 120 (GPR120), also called free fatty acid receptor 4 (FFAR4), is a member of the recently discovered G protein-coupled receptor family that can interact with both long-chain saturated fatty acids (C14-C18) and long-chain unsaturated fatty acids (C16-C22). Research shows that GPR120 regulates processes in different types of bone cells, directly or indirectly affecting bone metabolism. Our research reviewed the literature on the effects of GPR120 on bone marrow mesenchymal stem cells (BMMSCs), osteoblasts, osteoclasts, and chondrocytes, focusing on the research findings regarding the mechanism by which GPR120 alters specific bone metabolic diseases-osteoporosis and osteoarthritis. The data reviewed here provide a basis for clinical and basic research into the role of GPR120 on bone metabolic diseases.

## 1. Introduction

Bone metabolism refers to the interaction between erythropoietic and stromal cells in the bone marrow as well as the autologous interactions among bone cells, which drive bone remodeling processes [1]. Bone is always in a dynamic balance between bone formation and bone resorption. In this process, osteoblasts and osteoclasts are the leading players [1]. Osteoblasts synthesize the matrix of bone, while osteoclasts perform bone destruction and resorption. Other cells, such as bone marrow mesenchymal stem cells (BMMSCs), osteocytes, and chondrocytes, play essential roles in bone homeostasis. Once the dynamic balance of bone formation/resorption is disrupted, bone formation and healing dysfunction result in bone metabolic diseases such as osteoporosis [1-3]. Low bone mass and bone tissue microarchitecture degeneration are the main pathological features of osteoporosis which to increased bone fragility and fracture susceptibility and even death. According to reports, more than 200 million people in China have osteoporosis, and the average prevalence of this condition among the elderly is about 15.7%; osteoporosis in women is as high as 80%, especially in postmenopausal women [4, 5]. Osteoarthritis is another common bone metabolism disease; its incidence in adults is more than 10%. Chronic inflammation of articular cartilage and collagen degradation is its main pathogenic features [6, 7]. Metabolic bone diseases have become a major medical and socioeconomic challenge [4].

GPR120 interacts with both long-chain saturated fatty acids (C14-C18) and long-chain unsaturated fatty acids (C16-C22) [2, 8]. A lipid ligand binds to a GPR, causing a conformational change in the receptor and activation of intracellular guanine nucleotide binding proteins (G proteins). Depending on the type of G protein coupled to the receptor, distinct enzymes include stimulation of adenylate cyclase and guanylyl cyclase, phospholipase A2 and C, phosphodiesterase, and phosphatidylinositol 3 kinases (PI3Ks), which trigger different downstream signal pathways. FAs are important regulators of intestinal sex hormone secretion by activating GPRs. These hormones regulate gastrointestinal movement and secretion, insulin secretion, and modulate food intake [9].

Fatty acids are critical in bone metabolism [2]. The effects of fatty acids on bone metabolism can be either direct or indirect [10]. Studies have shown that GPR120 is expressed throughout the body, including in different types of osteocytes, and has been confirmed to be an essential target of various types of fatty acids that regulate bone metabolism [11, 12]. Fatty acid regulation of bone metabolism has recently been a research hotspot [2]. However, reviews on the mechanisms of GPR120 affecting bone metabolism are still largely missing. Our research reviewed the literature on the role of GPR120 in BMMSCs, osteoblasts, osteoclasts, and chondrocytes, and discussed the mechanism by which GPR120 regulates osteoporosis and osteoarthritis, to provide data to guide the clinical treatment of bone metabolic diseases.

## 2. GPR120 regulates bone mesenchymal stem cells

### 2.1 Selection of “adipogenic” or “osteogenic” differentiation of bone mesenchymal stem cells

A pluripotent stem cell, BMMSC, may differentiate into osteoblasts, chondrocytes or adipocytes [8]. On the other hand, bone formation mainly depends on BMMSCs and their differentiated osteoblasts [13]. Osteoblasts and bone marrow adipocytes originate from the same BMMSCs. This means that they differentiate into one cell line at the expense of the other [13]. As a result, decreased osteoblast differentiation or the enhanced ability of BMMSCs to differentiate into adipocytes will reduce the ratio of osteoblasts/adipocytes, resulting in increased fat in the bone marrow cavity and a significant decrease in osteogenic ability, leading to osteoporosiss [14, 15]. Marrow adipose tissue (MAT) differs from other fat depots in its properties and functions, and it plays a dynamic role in influencing bone mass and quality [16]. It is not only bone mineral density (BMD) and bone microarchitecture that determine bone strength, but also its microenvironmental tissues as well, such as bone marrow adipose tissue [16]. The nature and function of MAT are different from those of other fat banks, and this tissue influences bone mass and quality. Bone marrow is a dynamic organ, and the content and composition of MAT are regulated by hormonal stimulation, excess nutrition and deficiency [16]. There are many local interactions between bone marrow adipocytes and the surrounding microenvironment. In humans, variations in the number of bone marrow adipocytes in the distal tibia and proximal femur demonstrate that bone marrow adipocytes in the distal and proximal tibia have different characteristics, indicating that the composition of bone marrow adipocytes varies with location. MAT was negatively correlated with the overall and microstructure parameters of the distal tibia [16]. Compared with lumbar MAT, MAT at the distal tibia is less dynamic and highly regulated by hormones and changes in nutritional environment [16]. Fat cells in MAT produce fat factors that may have different paracrine and endocrine functions [16]. For example, adiponectin produced by bone marrow adipocytes participates in bone anabolism locally, but may have the opposite effect in the general body [16]. Mesenchymal stem cells in the bone marrow are more likely to respond to the subtle local changes of osteogenesis and fat stimulation [16]. Several cross-sectional studies using different measurement methods have shown that higher MAT is associated with vertebral fracture [17]. The increase in MAT and its lipotoxicity induce osteoclast formation, thereby causing bone marrow atrophy and bone loss in the elderly artificial blood [17]. It is believed that omega-3 fatty acids prevent stress on the endoplasmic reticulum and the activation of innate immune processes mediated by saturated fatty acids [18]. Supplement omega-3 fatty acids can decrease osteoclast production and osteoclast activity [18]. In addition, omega-3 fatty acids can decrease inflammation and lipotoxicity and reduce osteoclast production. omega-6 fatty acids may stimulate the uptake and storage of intracellular and extracellular fat, while omega-3 fatty acids may be able to compete with them and slow down the formation of fat and related lipotoxicity and inflammation in the bone marrow environment [18]. In mammals, a low dietary omega-3/omega-6 ratio will reduce bone formation and cause greater bone absorption [19]. In the aspect of indicating the activity of bone cells, the bone alkaline phase index plays a key role [20]. It was found that rats fed a diet high in omega-3 or a low ratio of omega-6 to omega-3 had higher serum bone alkaline phosphatase activities. The positive effects of omega-3 fatty acids on bone development are further supported[20]. Therefore, an intervention targeting the proliferation of bone marrow adipocytes can effectively treat chronic bone loss diseases such as osteoporosis [21].

### 2.2 GPR120 regulates fat metabolism

GPR120 is vital in physiological homeostasis, like adipogenesis, food preference, and appetite regulation [19]. In mice lacking GPR120, the adipocyte differentiation and adipogenesis are diminished and liver adipogenesis is enhanced on a high-fat diet, resulting in obesity, glucose intolerance, and fatty liver. In addition, insulin resistance in this type of mice is related to reduced insulin signal transduction and increased inflammation in adipose tissue [19]. There is high expression of GPR120 in adipose tissue, and it increases during adipocyte differentiation in mouse 3T3-L1 cells and human adipocytes, thus suggesting that this receptor is involved in the differentiation of adipocytes [22, 23]. GPR120 siRNA knockouts inhibit differentiation, supporting this hypothesis and indicating that GPR120 promotes the differentiation of 3T3-L1 cells and human adipocytes [23]. GPR120 gene expression was significantly reduced in islets of obese mice induced by a high-fat diet [24]. GPR120 agonists can improve glucose tolerance, reduce hyperinsulinemia and increase insulin sensitivity of insulin in obese mice fed high-fat diets [25]. Other studies have demonstrated that GPR120 and its downstream scaffold protein β-arrestin 2 display participating pairs of omega-3 fatty acids induced inhibition of inflammatory bodies [26]. EPA attenuates palmitic acid-induced 3T3-L1 adipocyte inflammatory gene expression, and NF-κB increased phosphorylation [27]. GPR120 silencing eliminated the anti-inflammatory effect of EPA. In the downstream signal analysis of GPR120, EPA reduced the increased expression of the TAK1/TAB1 complex induced by palmitic acid [27]. In addition, a diet containing EPA reduced p-JNK and phosphorylated p65 NF-κB, indicating that EPA exerts anti-inflammatory effects on 3T3-L1 adipocytes through GPR120 and alleviates the inflammation of adipose tissue in obese mice induced by diet [27]. MicroRNAs (miRNAs) are small non-coding RNAs that regulate diverse biological processes, like fat production [28]. MicroRNA-143 (miR-143) advances adipocyte differentiation and is associated with obese iron deficiency anemia in mice on a high-fat diet [28]. Studies have shown that the target of peroxisome proliferator-activated receptor γ (PPARγ) is miR-143. GPR120 knockdown in adipocytes reduced PPARγ and miR-143 expression [28]. Overexpression of GPR120 leads to increased expression of PPARγ and miR-143, while silencing of PPARγ inhibits GPR120-induced miR-143 [28].

In addition to brown adipose tissue (BAT)'s thermogenic activity, browning white adipose tissue also consumes energy [29]. Adults are stimulated to expend more energy when their BATs are activated [30]. GPR120 is greatly expressed in BAT and further increased in cold conditions [31]. Activation of the receptor with TUG-891 ((*4*-[(*4*-*Fluoro*-*4*′-*methyl*[1,1′-*biphenyl*]-*2*-yl)*methoxy*]-benzenepropanoic *acid*), a selective agonist, dramatically increased lipid oxidation and reduced the amount of fat in mice [31]. TUG-891 stimulated brown adipocytes to induce intracellular Ca^2+^ release, leading to mitochondrial depolarization and fission [31]. Therefore, activating GPR120 in BAT with TUG-891 and other ligands is a promising strategy to increase fat consumption [31]. Activating GPR120 promotes browning of white fat in mice and induces BAT activity, while GPR120 deletion promotes cold-induced browning [29]. There is also evidence that high consumption of omega-3 poly-unsaturated fatty acids or activation of the cell surface receptor GPR120 of omega-3 polyunsaturated fatty acids can stimulate adaptive thermogenesis [32]. 9-PAHSA, an endogenous mammalian lipid, and GPR120 ligand is anti-inflammatory and can enhance glucose uptake and inhibit the LPS/NF-κB pathway by activating GPR120 to induce adipocyte browning [33].

### 2.3 GPR120 regulates the generation and differentiation of BMMSCs

GPR120 has been proposed as the "button" that regulates the osteogenic and adipogenic differentiation of BMMSCs. *In vitro* studies in advanced humans showed that GPR120 expression level progressively raised in the process of BMMSCs osteogenic induction [34]. Knocking out GPR120 remarkably decreased osteogenic markers in BMMSCs, and osteogenic differentiation was significantly weakened [34]. TUG-891 is a highly selective agonist of GPR120, and induction with high doses of TUG-891 could not reverse the above results. Three mitogen-activated protein kinases (extracellular signal-related kinase (ERK), 1/2, p38 MAPK, and c-Jun N-terminal kinase (JNK)) protein kinase, MAPKs) signaling pathway is critical in osteoblast differentiation [34]. Further experiments showed that through the activation of integrin families (like integrin α1β1, α2β1, αvβ3) and the regulation of MAPK signals in a dose dependent manner, GPR120 can influence the process of osteogenic or adipogenic differentiation in BMMSCs [35]. A high concentration of TUG-891 activates GPR120 and up-regulates syntin α1, α2, β1, and the downstream Ras-ERK1/2 signaling pathway, which promotes the osteogenic differentiation of BMMSCs [35]. However, low concentrations of TUG-891 activate GPR120 to activate p38 through integrin αvβ3, inhibit ERK and induce adipogenesis [35]. TUG-891 dose-dependently modulates different integrin receptors through GPR120 [35]. *In vivo* studies showed that administering high doses of TUG-891 improved trabecular bone microstructure and increased the rate of bone formation in ovariectomized mice [35]. However, low-dose TUG-891 had no significant effect on mice, and the researchers believe that the reason needs further exploration [35].

It has also been shown that the differentiation of BMMSCs into osteoblasts or adipocytes involves substantial remodeling of the cytoplasmic membrane, a novel mechanism regulating the differentiation of BMMSCs [8]. Docosahexaenoic acid (DHA) is an omega-3 polyunsaturated fatty acid (PUFA) [36]. Osteoblast membranes are composed of DHA lipids component. GPR120 is a specific membrane receptor of DHA [35], which means that GPR120 can promote osteogenic differentiation by up-regulating the plasma membrane components of BMMSCs. However, no literature has determined whether DHA regulates the phosphorylation of Akt in the plasma membrane of bone marrow mesenchymal stem cells by means of GPR120, this regulates mesenchymal stem cell differentiation into osteoblasts from bone marrow.

### 2.4 GPR120 regulates apoptosis of BMMSCs

BMMSCs apoptosis may cause osteoporosis [14]. Studies show that GPR120 is vital in regulating the apoptosis of BMMSCs [35]. It has been reported that high doses of dexamethasone (Dex) can induce apoptosis and death of BMMSCs, resulting in osteoporosis [14]. Gao et al. found that ginsenoside-Rb2 can inhibit high-dose Dex-induced apoptosis of mouse BMMSCs via GPR120 [14]. Bone metabolism is becoming an area of interest due to the effectiveness of ginsenoside-Rb2 (Rb2), a 20(S)-protopanaxadiol glycoside extracted from ginseng, as a therapy for bone loss [14]. Rb2 dose-dependently increased the GPR120 expression in mouse BMMSCs and could reduce the activity of apoptotic factor caspase-3 through GRP120 [14]. In addition, under Dex-induced apoptosis of mouse BMMSCs, by means of GPR120, Rb2 may activate the Ras-ERK1/2 pathway in BMMSCs, and the anti-Dex-induced apoptosis of BMMSCs disappeared after ERK1/2 blockade [14]. Another experimental study found that GPR120, a specific membrane receptor for the omega-3 PUFAs eicosapentaenoic acid (EPA) [2]. The results of western blotting indicate that by activating GPR120 with TUG-891, pERK1/2 was increased, and knocking down GPR120 resulted in a significant reduction. Whereas as a result of the stimulus, neither ERK1/2, AKT, PI3K nor JNK expression levels were remarkably altered [35]. Therefore, GPR120 is expected to play an active role in preventing the apoptosis of BMMSCs induced by high-dose Dex.

## 3. GPR120 regulates osteoblasts

Derived from mesenchymal stem cells, osteoblasts are mononuclear cells [2]. Mineralized bone is formed by osteoblasts after absorption. The expression of Runt-associated transcription factor 2 (Runx2) is acknowledged as the earliest sign of osteoblast formation[11]. Runx2 up-regulates osteoblast-specific genes, like alkaline phosphatase (ALP), type 1 collagen α1 (COL1A1), and bone salivary protein (BSP)[11]. Differentiation of osteoclasts is regulated by receptor activators of nuclear factor kappa-B ligand (RANKL) and macrophage colony-stimulating factor (M-CSF) produced by osteoblasts. A decoy receptor for RANKL is further produced by osteoblasts, called osteoprotegerin (OPG). This prevents RANKL from binding to receptor activators of nuclear factor kappa-B (RANK) [11]. In such a manner, osteoblasts control osteoclast uptake and sustain a balance between uptake and formation [11]. Research has clarified that osteoblasts and their progenitors express GPR120, and GPR120 is upregulated in mature osteoblasts compared with their progenitors [37].

Ahn et al. showed that the GPR120 expressed by differentiated osteoblasts was more than that of undifferentiated osteoblasts when cultured *in vitro* [37]. Bone morphogenetic protein 2 (BMP-2) enhanced osteoblast differentiation [37], and the expression of GPR120 was further increased. They injected a concentration of 50 μM to 250 μM's DHA into the skull of mice and noted that the whole skull width of DHA treatment is wider than that of PBS treatment. DHA stimulates osteoblast activity through the expression of ALP and OCN mRNA. The knockout of GPR120 completely blocked the activity of osteoblasts stimulated by DHA [37].

Osteoblasts can produce OPG to prevent RANKL and RANK [8]. OPG is a protective agent against osteoclastogenesis and bone resorption [8]. Osteo-clastogenesis and bone resorption are enhanced by a low OPG/RANKL ratio, while osteoclastogenesis and bone resorption is inhibited by a high OPG/RANKL ratio [11]. In one of the studies by Kasonga et al., TUG-891, DHA, EPA, palmitic acid (PLA), and oleic acid (OA) all increased the expression of OPG in MC3T3-E1 cells via GPR120-β-arrestin 2 signaling pathway [11]. However, none of the above substances affected the expression of RANKL. RANKL expression remained unaffected by GPR120 or β-arrestin 2 knockdown, which theoretically would result in the raise of the OPG/RANKL ratio[11]. Nevertheless, TUG-891, DHA, and PLA remarkably raised the OPG/RANKL ratio in MC3T3-E1 cells via GPR120 and β-arrestin 2, but OA did not have a significant impact on OPG/RANKL ratio [11]. Kasonga et al. suggest that other signaling pathways may have roles [11]. Overly, GPR120 can bind different types of fatty acids and affect the metabolic activity of osteoblasts.

## 4. GPR120 regulates osteoclasts

Osteoclasts are multinucleated cells that develop from hematopoietic stem cells derived from the mononuclear-macrophage cell line [38]. The balance between bone resorption by osteoclasts and formation by osteoblasts is important for bone growth and fracture healing [38]. Abnormal activity of osteoclasts will upset this balance, and accelerated bone resorption will lead to osteoporosis [38]. Several studies have shown that GPR120 is highly expressed in RAW264.7 mouse precursor osteoclasts [25-27]. GPR120 can regulate bone metabolism by regulating various processes of osteoclast generation, apoptosis, and bone resorption [39].

### 4.1 GPR120 regulates osteoclastogenesis and differentiation of osteoclasts

The generation and differentiation of osteoclasts are regulated by many cytokines [22]. When bone marrow-derived macrophages (BMM) were exposed to M-CSF and RANKL, they differentiated into osteoclasts [40]. Precursor osteoclasts are proliferated, differentiated, and survive as a result of M-CSF, and it is possible to initiate TRAF6 conduction by combining RANKL and RANK [41]. The combination subsequently activates downstream signaling molecules such as NF-κB, Akt, MAPKs, inhibitor kappa B kinase β (IKKβ), and TAK1 kinase and ultimately activates c-FOS and nuclear factor of activated T-cells cytoplasmic 1 (NFATc1), which are master transcription factors for osteoclastogenesis [34, 38, 39, 42]. This series of processes stimulate osteo-clastogenesis and prevent osteoclast apoptosis [34].

Although previous works of literature have demonstrated the important role of GRP40 on osteoclasts [43, 44], some experiments suggest that GPR120 rather than GPR40 may regulate osteoclastogenesis [37, 39, 45]. GPR120 is greatly expressed in the RAW264.7 macrophage cell line [45]. Ahn et al. and Kim et al. showed that GPR120 is more expressed in mature osteoclasts than in its precursor cells. With the differentiation process of RAW264.7 cells, the expression of GPR120 was increased [34, 36], but GPR40 was hardly detected during this process [39]. Another study showed that in RAW264.7 cells, the expression of GPR120 was 100-fold higher than that of GPR40 [45], indicating that GPR120 might be critical in osteoclast differentiation.

The study by Kim et al. showed that the GPR120 agonist GW9508 significantly inhibited osteoclasto-genesis in a dose-dependent manner upon induction of BMM using RANKL and M-CSF[39]. GW9508 is the agonist of GPR120 [39]. Activation of GPR120 by GW9508 strongly inhibits NFATc1 and its downstream target genes and downstream signaling of RANKL[39]. Previously, GPR120 knockout alleviated the effect of GW9508 on the NFATc1 pathway and partially blocks RANKL signaling. However, different doses of GW9508 did not affect the generation and survival of BMM treated with M-CSF[39]. Different fatty acids also act directly on osteoclasts through GPR120. The omega-3 poly-unsaturated fatty acids DHA and EPA, as well as the omega-7 fatty acid PLA and the GPR120 agonist TUG-891 can reduce the number of osteoclasts through the GPR120-β-arrestin 2 pathway and inhibit the downstream signaling cascades of RANKL, p38, JNK, and ERK phosphorylation. In addition, they inhibit the nuclear translocation of NF-κB in osteoclasts and inhibit osteoclast differentiation [11]. Cornish et al. showed that long-chain saturated fatty acids with a chain length of C14-18 inhibited osteoclastogenesis by activating GPR120. However, polyunsaturated fatty acids such as omega-3 and omega-6 covering more carbon atoms did not inhibit osteoclastogenesis [45]. This may be because omega-3 and omega-6 polyunsaturated fatty acids activate GPR120 and PPARγ, which promotes osteoclast formation [45].

Classical pro-inflammatory factors, like interleukin-6 (interleukin-6, IL-6), interleukin-1 (interleukin-1, IL-1), tumor necrosis factor-alpha (tumor necrosis factor alpha, TNF-α) are essential mediators in osteoclast differentiation and bone resorption process *in vitro* and *in vivo* [46, 47]. IL-6 and IL-1 can stimulate osteoclasto-genesis and directly or indirectly affect bone turnover [48]. Additionally, TNF-α induces osteoclasto-genesis *in vitro* and *in vivo* [49]. Moreover, it has been proven that the direct inhibition of DHA on RANKL and TNF-α-induced precursor osteoclast differentiation by activating GPR120 [49]. The AH7614 reverses this effect in a dose-dependent manner [49]. Lipopolysaccharide (LPS) and Prevotella lipopoly-saccharide (Porphyromonas gingivalis lipopoly-saccharide, Pg-LPS) can induce inflammatory responses and can be used to induce bone loss models [50-52]. In RAW264.7 cells, activation of GPR120 by GW9508 remarkably inhibited LPS-induced phosphorylation of IκB kinase and JNK. Additionally, the activation inhibited the secretion of TNF-α and IL-6, whereas the effects of GW9508 in inhibiting the inflammatory cascade disappeared after GPR120 knockout [53]. In another study, it was revealed that in RAW264.7 cells, GPR120 combined with poly-unsaturated fatty acid 10-oxo-trans-11-octadecenoic acid (KetoC) inhibited Pg-LPS-induced TNF-α, IL- 6 and interleukin-1 beta (IL-1β) expression, through the NF-κB p65 pathway [54]. GPR120 also acts as a receptor for DHA and participates in anti-inflammatory processes *in vitro* and *in vivo*, thereby regulating bone metabolism [35]. The experiments by Kishikawa et al. showed that the number of osteoclasts and the ratio of bone resorption pits in the LPS-DHA combined administration group were remarkably lower in comparison to that in the LPS administration group [49]. The RANKL and TNF-α expression significantly decreased in mouse osteoclasts, both of which were blocked by GPR120 antagonists [49]. Similarly, in GPR120 knockout mice, the inhibitory effects of DHA on LPS-induced osteoclastogenesis and TNF-α, as well as RANKL, were also abolished [49]. The above studies indicate that GPR120 is crucial in anti-inflammatory-induced osteoclast formation and bone destruction.

Clinical and basic studies have shown that gut microbiota can affect bone metabolism [55]. The gut microbiota refers to the intestinal commensal, symbiotic, and pathogenic microorganisms [56]. The gut microbiota influences homeostasis of the skeletal system by affecting host metabolism, immune function, hormone secretion, and other pathways [56]. Disturbance in the gut microbiota can cause or exacerbate bone destruction [57, 58]. Britton et al. showed that *Lactobacillus reuteri* 6475 supernatants (CCS) inhibited osteoclastogenesis in RAW264.7 cells *in vitro* [59]. Further studies showed that CCS inhibited osteoclastogenesis in a concentration-dependent manner, and this inhibiting effect was more notable in the early stages of osteoclast differentiation. GPR120 antagonists entirely blocked the inhibitory effect of CCS on osteoclastogenesis, indicating that GPR120 targets CCS to inhibit osteoclastogenesis. Lactobacillus acid contained in *Lactobacillus reuteri* 6475 is a long-chain fatty acid [12]. However, when lactobacillus acid was used alone, the anti-osteoclastogenic effect of RANKL-induced osteoclasts was not entirely blocked by GPR120 antagonists. It is hypothesized that it contains additional target receptors or is affected by the concentration of lactobacillus acid treatment [12].

### 4.2 GPR120 regulates osteoclast apoptosis

The precursor and mature osteoclasts apoptosis are indispensable factors that control osteoclast number and bone resorption [39]. The study by Philippe et al. showed that high doses of GW9508 induced oxidative stress and apoptosis in the mitochondria of RAW264.7 precursor cells [60]. Although GW9508 can activate both GPR120 and GPR40 receptors, it has a higher affinity for GPR40 receptors. GW9508 affected the viability of RAW264.7 precursor cells after GPR40 knockout, making them less viable. Researchers have postulated that high doses of GW9508 trigger oxidative stress and apoptosis in RAW264.7 precursor cells, possibly through the GPR120 receptor [60]. Kim et al. showed that GW9508 promoted apoptosis of mature osteoclasts in a dose-dependent manner after M-CSF induction of mature osteoclasts [39]. During this process, apoptosis factors caspase-3 and caspase-9 are activated, and the expression of B-cell leukemia-2 (Bcl-2) family protein Bim is up-regulated [39]. The expression level of Bim is crucial for regulating osteoclast apoptosis, and the survival rate of Bim-deficient osteoclasts is increased [39]. Knockout of GPR120 abrogated the above apoptosis effect of mature osteoclasts [39]. However, the GPR120 activation did not influence the apoptosis of M-CSF-treated BMM cells suggesting that GPR120 is an important regulator of osteoclast apoptosis [39].

### 4.3 GPR120 regulates osteoclast cytoskeleton organization and bone resorption

Mature osteoclasts possess bone resorption activity, and their bone resorption process is triggered within the seal zone in the actin ring; differentiated osteoclasts attach to the bone surface and secrete proteolytic enzymes that degrade and dissolve bone mineral and collagen matrices [41, 61]. The function of osteoclasts is influenced by the actin cytoskeleton, which is an essential organelle to osteoclasts-regulated bone resorption process [42]. While integrin β3 is a crucial regulator of osteoclast cytoskeleton organization, studies have shown that its absence results in osteoclast cytoskeleton loss, impaired bone resorption, and increased bone density [42, 62]. M-CSF induction restores the actin ring of osteoclasts, and M-CSF induces remodeling of osteoclast cytoskeleton organization by the signaling pathway shared by integrin β3. Studies have shown that GPR120 regulates the cytoskeletal polarization of osteoclasts [27]. Activation of GPR120 by GW9508 inhibited actin ring remodeling induced by M-CSF, and the integrin β3 expression. GPR120 knockout abolished the above effects [27]. However, GW9508 did not inhibit actin ring remodeling induced by RANKL [27]. The above findings suggest that the GPR120 activation concretely inhibits M-CSF-induced osteoclast cytoskeletal organization and afterward blocks bone resorption [27]. *In vivo* experiments by Kishikawa et al. showed that GPR120 mediated the inhibitory effect of DHA on LPS-induced osteoclast bone resorption[49]. Correspondingly, when GPR120 was knocked out in mice, DHA's inhibiting effect on LPS-induced osteoclastic bone resorption was suppressed. The discoveries illustrated that GPR120 is involved in osteoclast bone resorption inhibition [49].

## 5. GPR120 regulates chondrocytes

Chondrocytes are the primary type of chondrocytes existing in the cartilage stroma and cartilage lacuna [2]. They are differentiated from BMMSCs and can produce a cartilage extracellular matrix (ECM) mainly composed of proteoglycan and collagen [2]. Fatty acids are mainly integrated into chondrocytes in the form of phosphatidylcholine and triacylglycerol, and then the downstream signal pathway is mediated by receptors expressed on the chondrocyte membrane, such as GPR40 and GPR120 [2]. Chronic inflammation and type II collagen degradation of articular cartilage can lead to osteoarthritis [6, 7]. Studies have shown that GPR120 is expressed on the chondrocyte membrane and can mediate downstream signaling pathways together with other receptors, thereby affecting the physiological and pathological activities of chondrocytes [2]. The study of Koren et al. showed that DHA and EPA could promote the proliferation and differentiation of the ATDC5 chondrocyte cell line, and GPR120 is expressed in all differentiation stages of chondrocytes [63]. However, the expression level depends on the differentiation stage of cells, and its role is not yet fully understood [63]. Studies showed that IL-6 inhibited type II collagen production and increased the expression of catabolic enzymes in chondrocytes, whereas IL-8 promotes the production of degradative enzymes. In ATDC5 chondrocytes, GPR120 agonists attenuated inflammatory responses by inhibiting IL-1β-induced IL-6 and IL-8 expression and transcription factor NF-κB expression [64]. The SOX9 transcription factor plays a critical role in the production of type II collagen and proteoglycans. Activation of GPR120 can upregulate the SOX9 expression and increase type II collagen and the number of aggregated proteins which prevents IL-8-induced chondrocyte destruction [64].

**Figure 1.**
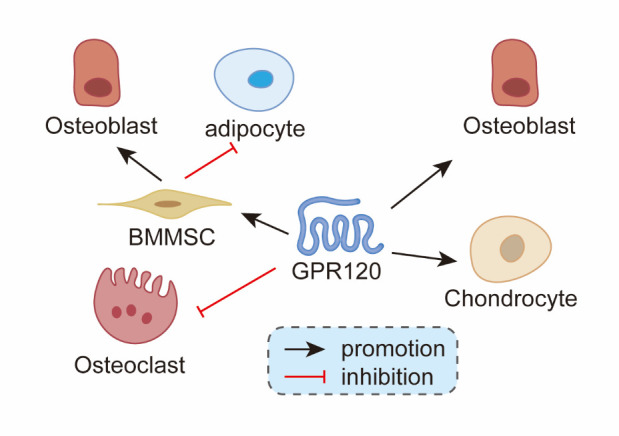
Effect of GPR120 on different types of bone cells.

Most reports suggest that omega-3 PUFAs protect against bone and joints. Conversely, omega-6 PUFAs are more likely to damage cartilage. Zhang et al. showed that cartilage thickness increased and TNF-α levels in serum and cartilage decreased in mice fed with a low omega-6/omega-3 PUFAs ratio [65]. The degradation of ECM arises from matrix metalloproteinase (MMP) or a disintegrin and metalloproteinase with thrombospondin motifs (ADAMTS) produced by chondrocytes, synovial fibroblasts, and macrophages [6]. Edible oils with a low omega-6/omega-3 PUFAs ratio can activate GPR120, thereby inhibiting the NF-κB pathway and its downstream matrix metalloproteinase 13 (MMP13) and aggrecan 5 (a disintegrin and metalloprotease with thrombospondin motifs 5, ADAMTS5) [65], protects cartilage cells and can effectively treat osteoarthritis [66]. In addition, osteoarthritis is associated with osteoporosis [7]. It has been reported that osteoarthritis is related to osteoclast-related receptors [7]. By blocking the binding of osteoclast-related receptors to ligands, it can reduce chondrocyte apoptosis and prevent osteoarthritis [7]. The study by Dai et al. used a dual model of osteoporotic osteoarthritis in mice [67]. Research elucidates that the low ratio omega-6/omega-3 PUFAs can activate GPR120 to inhibit the NF-κB signaling pathway, reduce the inflammatory response in osteoporotic osteoarthritis double model mice, and prevent the loss of proteoglycan in articular cartilage, significantly improving the articular cartilage structure [67]. Collectively, GPR120 acts directly or indirectly as a fatty acid receptor against chondrocyte inflammation.

## 6. Other mechanisms by which GPR120 regulates bone metabolism

Recent studies have shown that oxidative stress is one of the essential factors leading to the decline of cell function [68]. An increase in oxidative stress and lipid peroxidation is associated with bone loss as we age [69]. Previous studies have shown that GPR120 has antioxidant effects in human aortic endothelial cells and mouse adipose tissue [70, 71]. Regarding bone metabolism, reactive oxygen species (ROS) regulate various signaling pathways during osteogenesis, including Wnt/β-catenin, MAPK, and other pathways [72]. The production of ROS is also critical for RANKL-mediated osteoclast formation. RANK can increase ROS production by binding to RANKL [73]. Cheshmehkani et al. were the first to link GPR120 to the regulation of ROS in macrophages [74]. Phorbol-12-myristate 13-acetate (PMA) promotes the rapid production of endogenous ROS in cells [74]. Cheshmehkani et al. showed that both isoforms of GPR120 reduced PMA-induced ROS production in RAW264.7 cells, and one of the isoforms was also resistant to LPS-induced inflammatory responses[74]. Recent research findings have shown that activation of GPR120 by TUG-891 stimulates the nuclear factor E2-related factor 2 (Nrf2) in RAW264.7 cells to resist oxidation and reduce ROS secretion. This results in inhibition of the RANKL-induced osteoclast formation and bone resorption [73].

**Table 1:** Effects of different doses of GPR120 receptor agonists on different types of osteocytes.

Agonists	Subjects	Treatment, dosage	Results	Refs
GW9508	BMMSCs	RANKL+M-CSF, 25, 50, 100μM	Dose-dependently inhibits osteoclast formation	[39]
	Mature osteoclast	(1) M-CSF, 50, 100μM (2) RANKL+M-CSF, 100μM	(1) increases apoptosis and inhibits actin ring remodeling (2) regulates integrin and inhibits bone resorption function	
	RAW264.7	PMA, 1μM	Increases PMA-induced ROS production	[73]
		(1) 1, 10, 50μM (2) 100μM	(1) No effect on cell proliferation (2) Induce mitochondrial oxidative stress, apoptosis, and inhibit cell proliferation	[60]
	ATDC5 chondrocytes	IL-1β, 50μM	Reduces IL-1β-induced inflammatory cytokines, prevents the degradation of type II collagen and aggrecan, and inhibits NF-κB expression	[64]
	HAECs	Ox-LDL, 50μM	Improves HAECs vitality, ameliorates ox-LDL-induced oxidative stress, decreases the expression of pro-inflammatory cytokines, inhibits the expression of VCAM-1 and E-selectin, inhibits the adhesion of THP-1 cells and HAECs, reversed KLF2 reduction	[70]
TUG891	BMMSCs	(1) osteogenic medium, 30, 50, 100μM (2) osteogenic medium, 50μM (3) osteogenic medium, 5μM (4) osteogenic medium, 0.5μM (5) osteogenic medium, 0.1, 0.5μM	(1) activates the Ras/ERK1/2 pathway; inhibits p38 phosphorylation, and promotes osteogenic differentiation (2) regulates the integrin family to promote osteogenic differentiation (3) significantly inhibits adipogenesis (4) regulates the integrin family to inhibit osteogenesis and promote adipogenicity (5) promotes the phosphorylation level of p38, inhibits the phosphorylation of ERK1/2, inhibits osteogenesis, and promotes adipogenic differentiation	[36]
		Dex, 10μM	Significantly inhibits the apoptotic effect of Dex and improves the survival rate of BMMSCs	[14]
	MC3T3-E1 precursor osteoblasts	Osteogenic medium, 100μM	Increases cell viability, expression of osteogenic genes, increases OPG and OPG/RANKL ratio	[11]
	RAW264.7 cells	RANKL, 20, 40, 60, 80, 100μM	(1) 40-100μM: significantly reduces the formation of osteoclasts (2) 80-100μM: significantly reduces the bone resorption pit area (3) 100μM: increases the nuclear translocation of Nrf2 and the expression of HO-1 and NQO1, inhibits the expression of Nox1, and significantly reduces the percentage of ROS-positive cells	[73]
		PMA, 3μM	Reduces PMA-induced ROS production	[74]
	ATDC5 chondrocytes	IL-1β, 10μM	Significantly prevents IL-1β-induced reduction of type II collagen and aggregated proteins	[64]
	Ovariectomized mice	(1) local direct injection of TUG-891 into the bone marrow cavity of mice, 10, 30, 50μM/kg/d (2) local direct injection of TUG-891 into the bone marrow cavity of mice, 0.1, 1μM/kg/d	(1) increases bone mass and improves the microstructure of trabecular bone in the distal femur, increases the rate of bone formation (2) does not improve bone microstructure	[36]
	HAECs	Ox-LDL, 10μM	Improves the vitality of HAECs, ameliorates ox-LDL-induced HAECs oxidative stress, decrease the expression of pro-inflammatory cytokines, inhibits the expression of VCAM-1 and E-selectin, inhibits the adhesion of THP-1 cells and HAECs, reversed KLF2 reduction	[70]

Annotation: BMMSCs, bone marrow mesenchymal stem cells; Dex, dexamethasone; E-selectin, endothelial selectin; HAECs, human aortic endothelial cells; HO-1, heme oxygenase-1; IL-1β, interleukin-1 beta; KLF2, Krüppel-like factor 2; M-CSF, macrophage colony-stimulating factor; Nox1, nicotinamide adenine dinucleotide phosphate (NADPH) oxidase; NQO1, NADPH: quinone reductase; Nrf2, nuclear factor E2-related factor 2; OPG, osteoprotegerin; ox-LDL, oxidized low-density lipoprotein; PMA, phorbol-12-myristate 13-acetate; RANKL, receptor activators of nuclear factor kappa-B ligand; ROS, reactive oxygen species; VCAM-1, vascular cellular adhesion molecule-1.

**Figure 2.**
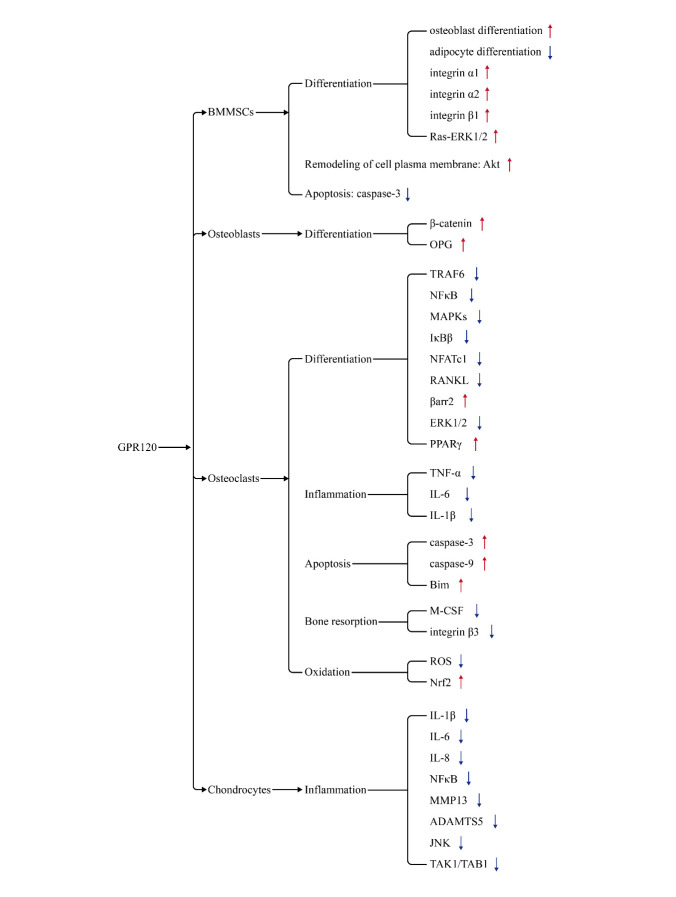
Pathway map of GPR120 on different types of osteocytes. The sign (-) means promotion, the sign (-) means inhibition.

The interplay between obesity and bone metabolism is complex. Historically, obesity has been considered a protective measure against osteoporosis [10, 75]. However, after statistical adjustment, the relationship between fat mass and bone mass was negative, while the relationship between fat mass and fracture was positive. This suggests that excessive obesity deteriorates bone health [10, 75]. Obese patients show upregulation of proinflammatory cytokines, leading to low-grade systemic inflammation, whereas some systemic inflammatory diseases can lead to increased bone resorption [75, 76]. In mice and humans, the expression of deficiency in GPR120 gene expression results in obesity and insulin resistance. Activation of GPR120 induces M2 anti-inflammatory phenotype macrophage polarization and inhibits macrophage migration into adipose tissue [77]. GPR120 is a physiological receptor for omega-3 fatty acids in macrophages and adipocytes, mediating potent anti-inflammatory and insulin-sensitizing effects [53]. In response to LCFA stimulation, GPR120 may influence gastrointestinal and pancreatic hormone secretion directly or indirectly. It can also influence lipid and/or glucose metabolism in adipose tissues, livers, and muscles [78]. In addition, in adipose tissue and macrophages, GPR120 is believed to mediate anti-inflammatory and insulin-sensitizing effects [78]. Long-chain fatty acids, in particular palmitoleic acid (PA), and the omega-3 fatty acids, α-linolenic acid (ALA), DHA and EPA have established activators of GPR120 [53, 79, 80]. Some studies have shown that the stimulation of these fatty acids on GPR120 will increase the secretion of glucagon-like peptide-1 (GLP-1) *in vitro* and *in vivo*, thus increasing the circulating insulin level [79]. The stimulation of GPR120 by ALA and PA increased the secretion of cholecystokinin *in vivo* and in mouse intestinal endocrine cells [81]. The findings of these studies indicate that GPR120 agonists can be used to treat obesity, type 2 diabetes (T2DM) and other metabolic disorders [78].

In conclusion, the activation of GPR120 reduces the oxidative stress response in different tissues and cells, as well as insulin resistance and inflammatory response, which will help reduce the occurrence of bone metabolic diseases and the antioxidant effect of GPR120 in osteocytes forms a foundation for future research. The effects of different doses of GPR120 receptor agonists and inhibitors on various types of osteocytes are shown in Tables 1 and 2. The effect of GPR120 on different types of bone cells is shown in Figure 1 and 2.

**Table 2:** Effects of different doses of GPR120 receptor inhibitors on different types of osteocytes.

Inhibitors	Subjects	Treatment, dosage	Results	Refs
AH7614	RAW264.7	RANKL +*Lactobacillus reuteri*, 1 μM	completely abolishes *Lactobacillus reuteri*’s ability to inhibit osteoclast formation	[12]
		(1) PMA, 1μM (2) PMA+TUG-891, 3 μM, μM (3) PMA+DHA, 1 μM	(1) increases PMA-induced ROS production (2) blocks the ability of TUG-891 to inhibit PMA-induced ROS production (3) reverses the effect of DHA on PMA-induced ROS production	[73]
		M-CSF+RANKL/TNF-α+DHA, 1, 10, 100, 1000 ng/ml	dose-dependently increases the number of osteoclasts	[49]
	C57/bl6 mice	Calvarial injection of AH7614 (100 μg/day) in LPS+DHA mice	attenuates the inhibitory effect of DHA on LPS-induced osteoclastogenesis, decrease in CTX levels, bone resorption, RANKL and TNF-α expression	

Annotation: CTX, C-terminal telopeptide; DHA, docosahexaenoic acid; LPS, lipopolysaccharide; M-CSF, macrophage colony-stimulating factor; PMA, phorbol-12-myristate 13-acetate; RANKL, receptor activators of nuclear factor kappa-B ligand; ROS, reactive oxygen species; TNF-α, tumor necrosis factor-alpha.

## 7. Summary

Recently, GPR120 has been a newly discovered G protein-coupled receptor in recent years, which is expressed in various types of osteocytes [11]. This review focuses on the regulatory effects of GPR120 on the metabolic activities of four types of osteocytes-BMMSCs, osteoblasts, osteoclasts, and chondrocytes. Studies have shown that GPR120 activation can directly or indirectly promote the osteogenic differentiation of BMMSCs and inhibit the apoptosis of BMMSCs, promote the formation and differentiation of osteoblasts and inhibit apoptosis of osteoblasts. Moreover, GPR120 activation inhibits the formation and differentiation of osteoclasts, promotes the apoptosis of mature osteoclasts, and inhibits the bone resorption function of osteoclasts; the above effects can prevent and treat osteoporosis. Fatty acids can affect bone metabolism [2]. To date, the majority of studies show that omega-6 fatty acids adversely affect bone metabolism, while omega-3 fatty acids positively affect bone metabolism [18]. GPR120 binds long-chain fatty acids and is a natural receptor for the omega-3 polyunsaturated fatty acids DHA and EPA[2, 36], mediating its direct effects on various osteocytes. In addition, GPR120 mediates the anti-inflammatory effect of omega-3 PUFAs on chondrocytes, thereby relieving osteoarthritis [64, 67]. In particular, we note that with the aging process, the level of "osteo-induced" fatty acids in the body gradually decreases, while the level of "fat-induced" fatty acids increases. After different doses of ligands-activated GPR120, BMMSCs show different osteogenic and adipogenic differentiation, respectively. This feature of ligand-dependent GPR120 may adjust the physiological process of BMMSCs [35]. In addition, since GW9508 is a co-agonist of GPR120 and GPR40 [39, 44], it is imperative to define the active receptor. Gene knockout or the use of GPR120-specific agonist TUG-891 are ideal experimental protocols.

In general, the prevailing literature demonstrates that GPR120 receptor has beneficial effects on bone metabolism. Notably, fatty acids not only serve as energy substrates in bone metabolism but also act as sources, structures, relative concentrations, and metabolic molecules with important effects on bone metabolism. However, the pros and cons of fatty acids on bone metabolism are still debatable [80, 81]. Therefore, it will be important to conduct more clinical prospective studies to determine whether GPR120, a fatty acid receptor, affects bone health in the long term.

## References

[1] Wang J, Lang X, Wang W, Zhang H, Zhang Y, Wang X (2021). Participation and regulatory mechanism of interleukin-1 during bone metabolism. Chinese Journal of Tissue Engineering Research, 25:5851-5858.

[2] Bao M, Zhang K, Wei Y, Hua W, Gao Y, Li X, et al. (2020). Therapeutic potentials and modulatory mechanisms of fatty acids in bone. Cell Prolif, 53:e12735. 31797479 10.1111/cpr.12735 PMC7046483

[3] Chen X, Wang Z, Duan N, Zhu G, Schwarz EM, Xie C (2018). Osteoblast-osteoclast interactions. Connect Tissue Res, 59:99-107. 28324674 10.1080/03008207.2017.1290085 PMC5612831

[4] Greenblatt MB, Tsai JN, Wein MN (2017). Bone Turnover Markers in the Diagnosis and Monitoring of Metabolic Bone Disease. Clin Chem, 63:464-474. 27940448 10.1373/clinchem.2016.259085 PMC5549920

[5] Feng Y, Zhu L, Gu Y, Wang LJ, Niu BJ, Cai F, et al. (2020). Association of Gremlin-2 gene polymorphisms with osteoporosis risk in Chinese postmenopausal women. Biosci Rep, 40. 10.1042/BSR20200554 PMC7182657 32297643

[6] Tateiwa D, Yoshikawa H, Kaito T (2019). Cartilage and Bone Destruction in Arthritis: Pathogenesis and Treatment Strategy: A Literature Review. Cells, 8. 10.3390/cells8080818 PMC6721572 31382539

[7] Park DR, Kim J, Kim GM, Lee H, Kim M, Hwang D, et al. (2020). Osteoclast-associated receptor blockade prevents articular cartilage destruction via chondrocyte apoptosis regulation. Nat Commun, 11:4343. 32859940 10.1038/s41467-020-18208-y PMC7455568

[8] Abshirini M, Ilesanmi-Oyelere BL, Kruger MC (2021). Potential modulatory mechanisms of action by long-chain polyunsaturated fatty acids on bone cell and chondrocyte metabolism. Prog Lipid Res, 83:101113. 34217732 10.1016/j.plipres.2021.101113

[9] Vinolo MAR, Hirabara SM, Curi R (2012). G-protein-coupled receptors as fat sensors. Current Opinion in Clinical Nutrition and Metabolic Care, 15:112-116. 22234165 10.1097/MCO.0b013e32834f4598

[10] Wauquier F, Leotoing L, Philippe C, Spilmont M, Coxam V, Wittrant Y (2015). Pros and cons of fatty acids in bone biology. Prog Lipid Res, 58:121-145. 25835096 10.1016/j.plipres.2015.03.001

[11] Kasonga AE, Kruger MC, Coetzee M (2019). Free fatty acid receptor 4-beta-arrestin 2 pathway mediates the effects of different classes of unsaturated fatty acids in osteoclasts and osteoblasts. Biochim Biophys Acta Mol Cell Biol Lipids, 1864:281-289. 30578965 10.1016/j.bbalip.2018.12.009

[12] Quach D, Parameswaran N, McCabe L, Britton RA (2019). Characterizing how probiotic Lactobacillus reuteri 6475 and lactobacillic acid mediate suppression of osteoclast differentiation. Bone Rep, 11:100227. 31763377 10.1016/j.bonr.2019.100227 PMC6864341

[13] de Araujo IM, Parreiras ESLT, Carvalho AL, Elias J Jr, Salmon CEG, de Paula FJA (2020). Insulin resistance negatively affects bone quality not quantity: the relationship between bone and adipose tissue. Osteoporos Int, 31:1125-1133. 32108240 10.1007/s00198-020-05365-5

[14] Gao B, Huang Q, Jie Q, Zhang HY, Wang L, Guo YS, et al. (2015). Ginsenoside-Rb2 Inhibits Dexamethasone-Induced Apoptosis Through Promotion of GPR120 Induction in Bone Marrow-Derived Mesenchymal Stem Cells. Stem Cells and Development, 24:781-790. 25314926 10.1089/scd.2014.0367

[15] Mori K, Suzuki K, Hozumi A, Goto H, Tomita M, Koseki H, et al. (2014). Potentiation of osteoclastogenesis by adipogenic conversion of bone marrow-derived mesenchymal stem cells. Biomed Res, 35:153-159. 24759183 10.2220/biomedres.35.153

[16] Singhal V, Flores LPT, Stanford FC, Toth AT, Carmine B, Misra M, et al. (2018). Differential associations between appendicular and axial marrow adipose tissue with bone microarchitecture in adolescents and young adults with obesity. Bone, 116:203-206. 30107255 10.1016/j.bone.2018.08.009 PMC6158042

[17] Li X, Schwartz AV (2020). MRI Assessment of Bone Marrow Composition in Osteoporosis. Curr Osteoporos Rep, 18:57-66. 31955352 10.1007/s11914-020-00562-x PMC9044504

[18] Bani Hassan E, Alderghaffar M, Wauquier F, Coxam V, Demontiero O, Vogrin S, et al. (2019). The effects of dietary fatty acids on bone, hematopoietic marrow and marrow adipose tissue in a murine model of senile osteoporosis. Aging (Albany NY), 11:7938-7947. 31553309 10.18632/aging.102299 PMC6781972

[19] Riera-Heredia N, Lutfi E, Gutierrez J, Navarro I, Capilla E (2019). Fatty acids from fish or vegetable oils promote the adipogenic fate of mesenchymal stem cells derived from gilthead sea bream bone potentially through different pathways. PLoS One, 14:e0215926. 31017945 10.1371/journal.pone.0215926 PMC6481918

[20] Cheng M, Xiang T, Wu YL, Jia L, Su Y, Feng JW (2022). Effect of Bone Marrow Mesenchymal Stem Cells on Mechanical Dynamics and BALP/CTX-1 Expression in Rats with Osteoporotic Vertebral Fracture. Sichuan Da Xue Xue Bao Yi Xue Ban, 53:815-820. 36224683 10.12182/20220960506 PMC10408791

[21] Muruganandan S, Ionescu AM, Sinal CJ (2020). At the Crossroads of the Adipocyte and Osteoclast Differentiation Programs: Future Therapeutic Perspectives. Int J Mol Sci, 21. 10.3390/ijms21072277 PMC7177886 32224846

[22] Ichimura A, Hirasawa A, Poulain-Godefroy O, Bonnefond A, Hara T, Yengo L, et al. (2012). Dysfunction of lipid sensor GPR120 leads to obesity in both mouse and human. Nature, 483:350-U149. 22343897 10.1038/nature10798

[23] Ulven T, Christiansen E (2015). Dietary Fatty Acids and Their Potential for Controlling Metabolic Diseases Through Activation of FFA4/GPR120. Annual Review of Nutrition, Vol 35, 35:239-263. 10.1146/annurev-nutr-071714-034410 26185978

[24] Zhao YF, Li XC, Liang XY, Zhao YY, Xie R, Zhang LJ, et al. (2020). GPR120 Regulates Pancreatic Polypeptide Secretion From Male Mouse Islets via PLC-Mediated Calcium Mobilization. Endocrinology, 161. 10.1210/endocr/bqaa157 32877513

[25] Oh DY, Walenta E, Akiyama TE, Lagakos WS, Lackey D, Pessentheiner AR, et al. (2014). A Gpr120-selective agonist improves insulin resistance and chronic inflammation in obese mice. Nat Med, 20:942-947. 24997608 10.1038/nm.3614 PMC4126875

[26] Yan YQ, Jiang W, Spinetti T, Tardivel A, Castillo R, Bourquin C, et al. (2013). Omega-3 Fatty Acids Prevent Inflammation and Metabolic Disorder through Inhibition of NLRP3 Inflammasome Activation. Immunity, 38:1154-1163. 23809162 10.1016/j.immuni.2013.05.015

[27] Yamada H, Umemoto T, Kakei M, Momomura S, Kawakami M, Ishikawa S, et al. (2017). Eicosapentaenoic acid shows anti-inflammatory effect via GPR120 in 3T3-L1 adipocytes and attenuates adipose tissue inflammation in diet-induced obese mice. Nutrition & Metabolism, 14. 10.1186/s12986-017-0188-0 PMC5422876 28503189

[28] Bae IS, Park PJ, Lee JH, Cho EG, Lee TR, Kim SH (2017). PPARgamma-mediated G-protein coupled receptor 120 signaling pathway promotes transcriptional activation of miR-143 in adipocytes. Gene, 626:64-69. 28495174 10.1016/j.gene.2017.05.016

[29] Quesada-Lopez T, Cereijo R, Turatsinze JV, Planavila A, Cairo M, Gavalda-Navarro A, et al. (2016). The lipid sensor GPR120 promotes brown fat activation and FGF21 release from adipocytes. Nat Commun, 7:13479. 27853148 10.1038/ncomms13479 PMC5118546

[30] Schilperoort M, van Dam AD, Hoeke G, Shabalina IG, Okolo A, Hanyaloglu AC, et al. (2018). The GPR120 agonist TUG-891 promotes metabolic health by stimulating mitochondrial respiration in brown fat. EMBO Mol Med, 10. 10.15252/emmm.201708047 PMC5840546 29343498

[31] Christian M (2020). Elucidation of the roles of brown and brite fat genes: GPR120 is a modulator of brown adipose tissue function. Experimental Physiology, 105:1201-1205. 32144819 10.1113/EP087877 PMC8650997

[32] Fan R, Koehler K, Chung S (2019). Adaptive thermogenesis by dietary n-3 polyunsaturated fatty acids: Emerging evidence and mechanisms. Biochimica Et Biophysica Acta-Molecular and Cell Biology of Lipids, 1864:59-70. 10.1016/j.bbalip.2018.04.012 PMC6197923 29679742

[33] Wang YM, Liu HX, Fang NY (2018). 9-PAHSA promotes browning of white fat via activating G-protein-coupled receptor 120 and inhibiting lipopolysaccharide / NF-kappa B pathway. Biochem Biophys Res Commun, 506:153-160. 30340828 10.1016/j.bbrc.2018.09.050

[34] Koga Y, Tsurumaki H, Aoki-Saito H, Sato M, Yatomi M, Takehara K, et al. (2019). Roles of Cyclic AMP Response Element Binding Activation in the ERK1/2 and p38 MAPK Signalling Pathway in Central Nervous System, Cardiovascular System, Osteoclast Differentiation and Mucin and Cytokine Production. Int J Mol Sci, 20. 10.3390/ijms20061346 PMC6470985 30884895

[35] Gao B, Huang Q, Jie Q, Lu WG, Wang L, Li XJ, et al. (2015). GPR120: A bi-potential mediator to modulate the osteogenic and adipogenic differentiation of BMMSCs. Sci Rep, 5:14080. 26365922 10.1038/srep14080 PMC4568495

[36] Kim H, Oh B, Park-Min KH (2021). Regulation of Osteoclast Differentiation and Activity by Lipid Metabolism. Cells, 10. 10.3390/cells10010089 PMC7825801 33430327

[37] Ahn SH, Park SY, Baek JE, Lee SY, Baek WY, Lee SY, et al. (2016). Free Fatty Acid Receptor 4 (GPR120) Stimulates Bone Formation and Suppresses Bone Resorption in the Presence of Elevated n-3 Fatty Acid Levels. Endocrinology, 157:2621-2635. 27145004 10.1210/en.2015-1855

[38] Yu SB, Kim HJ, Kang HM, Park BS, Lee JH, Kim IR (2018). Cordycepin Accelerates Osteoblast Mineralization and Attenuates Osteoclast Differentiation In Vitro. Evid Based Complement Alternat Med, 2018:5892957. 30410556 10.1155/2018/5892957 PMC6206560

[39] Kim HJ, Yoon HJ, Kim BK, Kang WY, Seong SJ, Lim MS, et al. (2016). G Protein-Coupled Receptor 120 Signaling Negatively Regulates Osteoclast Differentiation, Survival, and Function. J Cell Physiol, 231:844-851. 26280807 10.1002/jcp.25133

[40] Kodama J, Kaito T (2020). Osteoclast Multinucleation: Review of Current Literature. Int J Mol Sci, 21. 10.3390/ijms21165685 PMC7461040 32784443

[41] Kasonga AE, Deepak V, Kruger MC, Coetzee M (2015). Arachidonic acid and docosahexaenoic acid suppress osteoclast formation and activity in human CD14+ monocytes, in vitro. PLoS One, 10:e0125145. 25867515 10.1371/journal.pone.0125145 PMC4395026

[42] Kim HJ, Yoon HJ, Kim SY, Yoon YR (2014). A medium-chain fatty acid, capric acid, inhibits RANKL-induced osteoclast differentiation via the suppression of NF-kappaB signaling and blocks cytoskeletal organization and survival in mature osteoclasts. Mol Cells, 37:598-604. 25134536 10.14348/molcells.2014.0153 PMC4145371

[43] Philippe C, Wauquier F, Landrier JF, Bonnet L, Miot-Noirault E, Rochefort GY, et al. (2017). GPR40 mediates potential positive effects of a saturated fatty acid enriched diet on bone. Mol Nutr Food Res, 61. 10.1002/mnfr.201600219 27611773

[44] Wauquier F, Philippe C, Leotoing L, Mercier S, Davicco MJ, Lebecque P, et al. (2013). The free fatty acid receptor G protein-coupled receptor 40 (GPR40) protects from bone loss through inhibition of osteoclast differentiation. J Biol Chem, 288:6542-6551. 23335512 10.1074/jbc.M112.429084 PMC3585087

[45] Cornish J, MacGibbon A, Lin JM, Watson M, Callon KE, Tong PC, et al. (2008). Modulation of osteoclastogenesis by fatty acids. Endocrinology, 149:5688-5695. 18617622 10.1210/en.2008-0111

[46] Khosla S (2001). Minireview: the OPG/RANKL/RANK system. Endocrinology, 142:5050-5055. 11713196 10.1210/endo.142.12.8536

[47] Mundy GR (2007). Osteoporosis and inflammation. Nutr Rev, 65:S147-151. 18240539 10.1111/j.1753-4887.2007.tb00353.x

[48] Kupka T, Svoboda P, Bojkova M, Blaho M, Vasura A, Hrabovsky V, et al. (2020). Bone Metabolism in Inflammatory Bowel Diseases 2. Vnitr Lek, 66:432-436. 33380122

[49] Kishikawa A, Kitaura H, Kimura K, Ogawa S, Qi J, Shen WR, et al. (2019). Docosahexaenoic Acid Inhibits Inflammation-Induced Osteoclast Formation and Bone Resorption in vivo Through GPR120 by Inhibiting TNF-alpha Production in Macrophages and Directly Inhibiting Osteoclast Formation. Front Endocrinol (Lausanne), 10:157. 30949128 10.3389/fendo.2019.00157 PMC6436080

[50] Kim J, Lee H, Kang KS, Chun KH, Hwang GS (2015). Cordyceps militaris mushroom and cordycepin inhibit RANKL-induced osteoclast differentiation. J Med Food, 18:446-452. 25789604 10.1089/jmf.2014.3215

[51] Zhang Y, Yuan X, Wu Y, Pei M, Yang M, Wu X, et al. (2020). Liraglutide regulates bone destruction and exhibits anti-inflammatory effects in periodontitis in vitro and in vivo. J Dent, 94:103310. 32119967 10.1016/j.jdent.2020.103310

[52] Li X, Yu C, Hu Y, Xia X, Liao Y, Zhang J, et al. (2018). New Application of Psoralen and Angelicin on Periodontitis With Anti-bacterial, Anti-inflammatory, and Osteogenesis Effects. Front Cell Infect Microbiol, 8:178. 29922598 10.3389/fcimb.2018.00178 PMC5996246

[53] Oh DY, Talukdar S, Bae EJ, Imamura T, Morinaga H, Fan WQ, et al. (2010). GPR120 Is an Omega-3 Fatty Acid Receptor Mediating Potent Anti-inflammatory and Insulin-Sensitizing Effects. Cell, 142:687-698. 20813258 10.1016/j.cell.2010.07.041 PMC2956412

[54] Sulijaya B, Takahashi N, Yamada M, Yokoji M, Sato K, Aoki-Nonaka Y, et al. (2018). The anti-inflammatory effect of 10-oxo-trans-11-octadecenoic acid (KetoC) on RAW 264.7 cells stimulated with Porphyromonas gingivalis lipopolysaccharide. J Periodontal Res, 53:777-784. 29687443 10.1111/jre.12564

[55] He J, Xu S, Zhang B, Xiao C, Chen Z, Si F, et al. (2020). Gut microbiota and metabolite alterations associated with reduced bone mineral density or bone metabolic indexes in postmenopausal osteoporosis. Aging (Albany NY), 12:8583-8604. 32392181 10.18632/aging.103168 PMC7244073

[56] Behera J, Ison J, Tyagi SC, Tyagi N (2020). The role of gut microbiota in bone homeostasis. Bone, 135. 10.1016/j.bone.2020.115317 PMC8457311 32169602

[57] Rettedal EA, Ilesanmi-Oyelere BL, Roy NC, Coad J, Kruger MC (2021). The Gut Microbiome Is Altered in Postmenopausal Women With Osteoporosis and Osteopenia. JBMR Plus, 5:e10452. 33778322 10.1002/jbm4.10452 PMC7990138

[58] Lu L, Chen X, Liu Y, Yu X (2021). Gut microbiota and bone metabolism. FASEB J, 35:e21740. 34143911 10.1096/fj.202100451R

[59] Britton RA, Irwin R, Quach D, Schaefer L, Zhang J, Lee T, et al. (2014). Probiotic L. reuteri treatment prevents bone loss in a menopausal ovariectomized mouse model. J Cell Physiol, 229:1822-1830. 24677054 10.1002/jcp.24636 PMC4129456

[60] Philippe C, Wauquier F, Leotoing L, Coxam V, Wittrant Y (2013). GW9508, a free fatty acid receptor agonist, specifically induces cell death in bone resorbing precursor cells through increased oxidative stress from mitochondrial origin. Experimental Cell Research, 319:3035-3041. 23973666 10.1016/j.yexcr.2013.08.013

[61] Matsubara T, Kokabu S, Nakatomi C, Kinbara M, Maeda T, Yoshizawa M, et al. (2018). The Actin-Binding Protein PPP1r18 Regulates Maturation, Actin Organization, and Bone Resorption Activity of Osteoclasts. Mol Cell Biol, 38. 10.1128/MCB.00425-17 PMC5789032 29158294

[62] McHugh KP, Hodivala-Dilke K, Zheng MH, Namba N, Lam J, Novack D, et al. (2000). Mice lacking beta3 integrins are osteosclerotic because of dysfunctional osteoclasts. J Clin Invest, 105:433-440. 10683372 10.1172/JCI8905 PMC289172

[63] Koren N, Simsa-Maziel S, Shahar R, Schwartz B, Monsonego-Ornan E (2014). Exposure to omega-3 fatty acids at early age accelerate bone growth and improve bone quality. Journal of Nutritional Biochemistry, 25:623-633. 24746838 10.1016/j.jnutbio.2014.01.012

[64] Xu Z, Ke T, Zhang Y, Fu C, He W (2020). Agonism of GPR120 prevented IL-1beta-induced reduction of extracellular matrix through SOX-9. Aging (Albany NY), 12:12074-12085. 32580167 10.18632/aging.103375 PMC7343462

[65] Zhang T, Dai Y, Zhang L, Tian Y, Li Z, Wang J (2020). Effects of Edible Oils with Different n-6/n-3 PUFA Ratios on Articular Cartilage Degeneration via Regulating the NF-kappaB Signaling Pathway. J Agric Food Chem, 68:12641-12650. 33136410 10.1021/acs.jafc.0c05240

[66] Zhen G, Wen C, Jia X, Li Y, Crane JL, Mears SC, et al. (2013). Inhibition of TGF-beta signaling in mesenchymal stem cells of subchondral bone attenuates osteoarthritis. Nat Med, 19:704-712. 23685840 10.1038/nm.3143 PMC3676689

[67] Dai YF, Zhang L, Yan ZY, Li Z, Fu M, Xue CH, et al. (2021). A low proportion n-6/n-3 PUFA diet supplemented with Antarctic krill (Euphausia superba) oil protects against osteoarthritis by attenuating inflammation in ovariectomized mice. Food & Function, 12:6766-6779. 34160515 10.1039/d1fo00056j

[68] Almeida M, Han L, Martin-Millan M, Plotkin LI, Stewart SA, Roberson PK, et al. (2007). Skeletal involution by age-associated oxidative stress and its acceleration by loss of sex steroids. J Biol Chem, 282:27285-27297. 17623659 10.1074/jbc.M702810200 PMC3119455

[69] Palmieri M, Almeida M, Nookaew I, Gomez-Acevedo H, Joseph TE, Que X, et al. (2021). Neutralization of oxidized phospholipids attenuates age-associated bone loss in mice. Aging Cell, 20:e13442. 34278710 10.1111/acel.13442 PMC8373359

[70] Jiang T, Jiang D, You D, Zhang L, Liu L, Zhao Q (2020). Agonism of GPR120 prevents ox-LDL-induced attachment of monocytes to endothelial cells. Chem Biol Interact, 316:108916. 31870843 10.1016/j.cbi.2019.108916

[71] Amos D, Cook C, Santanam N (2019). Omega 3 rich diet modulates energy metabolism via GPR120-Nrf2 crosstalk in a novel antioxidant mouse model. Biochim Biophys Acta Mol Cell Biol Lipids, 1864:466-488. 30658097 10.1016/j.bbalip.2019.01.002 PMC6414231

[72] Wang F, Yin PP, Lu Y, Zhou ZB, Jiang CL, Liu YJ, et al. (2015). Cordycepin prevents oxidative stress-induced inhibition of osteogenesis. Oncotarget, 6:35496-35508. 26462178 10.18632/oncotarget.6072 PMC4742120

[73] Sithole C, Pieterse C, Howard K, Kasonga A (2021). GPR120 Inhibits RANKL-Induced Osteoclast Formation and Resorption by Attenuating Reactive Oxygen Species Production in RAW264.7 Murine Macrophages. Int J Mol Sci, 22. 10.3390/ijms221910544 PMC8508775 34638884

[74] Cheshmehkani A, Senatorov IS, Dhuguru J, Ghoneim O, Moniri NH (2017). Free-fatty acid receptor-4 (FFA4) modulates ROS generation and COX-2 expression via the C-terminal beta-arrestin phosphosensor in Raw 264.7 macrophages. Biochem Pharmacol, 146:139-150. 28943238 10.1016/j.bcp.2017.09.008 PMC5705417

[75] Gkastaris K, Goulis DG, Potoupnis M, Anastasilakis AD, Kapetanos G (2020). Obesity, osteoporosis and bone metabolism. J Musculoskelet Neuronal Interact, 20:372-381. 32877973 PMC7493444

[76] Lacativa PG, Farias ML (2010). Osteoporosis and inflammation. Arq Bras Endocrinol Metabol, 54:123-132. 20485900 10.1590/s0004-27302010000200007

[77] Im DS (2016). Functions of omega-3 fatty acids and FFA4 (GPR120) in macrophages. European Journal of Pharmacology, 785:36-43. 25987421 10.1016/j.ejphar.2015.03.094

[78] Zhang X, Macielag MJ (2020). GPR120 agonists for the treatment of diabetes: a patent review (2014 present). Expert Opin Ther Pat, 30:729-742. 32799609 10.1080/13543776.2020.1811852

[79] Hirasawa A, Tsumaya K, Awaji T, Katsuma S, Adachi T, Yamada M, et al. (2005). Free fatty acids regulate gut incretin glucagon-like peptide-1 secretion through GPR120. Nature Medicine, 11:90-94. 10.1038/nm1168 15619630

[80] Poulsen RC, Kruger MC (2006). Detrimental effect of eicosapentaenoic acid supplementation on bone following ovariectomy in rats. Prostaglandins Leukot Essent Fatty Acids, 75:419-427. 17029936 10.1016/j.plefa.2006.08.003

[81] Fu M, Tian Y, Zhang T, Zhan Q, Zhang L, Wang J (2020). Comparative study of DHA-enriched phosphatidylcholine and EPA-enriched phosphatidylcholine on ameliorating high bone turnover via regulation of the osteogenesis-related Wnt/beta-catenin pathway in ovariectomized mice. Food Funct, 11:10094-10104. 33140795 10.1039/d0fo01563f

